# Rapid acquisition of HPV around the time of sexual debut in adolescent girls in Tanzania

**DOI:** 10.1093/ije/dyv367

**Published:** 2016-03-04

**Authors:** Catherine F Houlihan, Kathy Baisley, Ignacio G Bravo, Saidi Kapiga, Silvia de Sanjosé, John Changalucha, David A Ross, Richard J Hayes, Deborah Watson-Jones

**Affiliations:** ^1^ Clinical Research Department, London School of Hygiene and Tropical Medicine, London, UK; ^2^ Mwanza Intervention Trials Unit, Mwanza, Tanzania; ^3^ MRC Tropical Epidemiology Group, London School of Hygiene and Tropical Medicine, London, UK; ^4^ Unit of Infections and Cancer, Institut Català d’Oncologia, Barcelona, Spain; ^5^ CIBER ESP, Barcelona, Spain; ^6^ National Institute for Medical Research, Mwanza, Tanzania

**Keywords:** Human papillomavirus, sexual debut, sub-Saharan Africa

## Abstract

**Background:** No reports exist on genotype-specific human papillomavirus (HPV) acquisition in girls after first sex in sub-Saharan Africa, despite high HPV prevalence and cervical cancer incidence.

**Methods:** We followed 503 HP-unvaccinated girls aged 15-16 years in Mwanza, Tanzania, 3-monthly for 18 months with interviews and self-administered vaginal swabs. Swabs were tested for 13 higHRisk and 24 low-risk HPV genotypes. Incidence, clearance and duration of overall HPV and genotype-specific infections were calculated and associated factors evaluated.

**Results** : A total of 106 participants reported first sex prior to enrolment ( *N* = 29) or during follow-up (N = 77). One was HIV-positive at the final visit. The remaining 105 girls contributed 323 adequate specimens. Incidence of any new HPV genotype was 225/100 person-years (pys), and incidence of vaccine types HPV-6, -11, -16 and -18 were 12, 2, 2 and 7/100 pys, respectively. Reporting sex in the past 3 months and knowing the most recent sexual partner for a longer period before sex were associated with HPV acquisition. Median time from reported sexual debut to first HPVinfection was 5 months, and infection duration was 6 months.

**Conclusion:** This is the first description of HPV acquisition after first sex in sub-Saharan Africa where the incidence of cervical cancer is amongst the highest in the world. HPV incidence was very high after first sex, including some vaccine genotypes, and infection duration was short. This very high HPV incidence may help explain high cervical cancer rates, and supports recommendations that the HPV vaccine should be given to girls before first sex.

Key Messages

- This is the first description of HPV acquisition after first sex in sub-Saharan Africa where the incidence of cervical cancer is amongst the highest in the world.
- HPV incidence was very high after first sex, including of vaccine genotypes.
- Duration of HPV infection was short in these adolescent girls.

## Introduction

A number of closely related human papillomavirus (HPV) genotypes are classified by the International Agency for Research on Cancers (IARC) as oncogenic (Group I) or probably oncogenic (Group IIA) ^1^ and are commonly referred to as ‘higHRisk’ (HR) HPVs. Persistent infection (repeated detection over at least 6 months) with HR-HPV is associated with ano-genital cancers in men and women. ^2^^,^^3^ Infection with HR-HPV genotypes is the primary cause of cervical cancer, ^4^ and the highest age-standardized cervical cancer incidence and mortality worldwide are seen in sub-Saharan Africa (SSA), along with some of the highest HPV prevalences. ^5^^,^^6^ Worldwide data have shown that the highest prevalence is in women under 25 years old. ^6^ From limited studies which have tested girls for HPV before and after first sex, prevalence is high following sexual debut. ^7–9^

Current HPV vaccines are prophylactic, not therapeutic, and should be given before HPV acquisition. ^10^ Knowledge of the rates and timing of HPV acquisition is thus essential to inform HPV vaccination policy. To date, no studies have documented genotype-specific HPV incidence or overall HPV incidence in girls in SSA around the time of sexual debut. A national HPV vaccination programme for Tanzania, although in the planning stages, has not yet commenced. In order to examine initial HPV infection and natural history, we enrolled 15- and 16-year-old unvaccinated girls and followed them 3-monthly for 18 months in Mwanza, Tanzania.

## Methods

### Cohort enrolment

The cohort was enrolled as described previously. ^11^ Briefly, for preparation for an HPV vaccination trial, registration lists of girls enrolled in government primary schools in three districts of Mwanza region, northern Tanzania, were collected in 2010. ^12^ We enrolled girls who had been in class 6 in 2010 in one of the 82 government schools not randomly selected for vaccination. Additional enrolment eligibility criteria included: being aged 15 or 16 years; self-reporting never having had sex and currently not pregnant; able to attend appointments; and willing to self-administer a vaginal swab. Since the enrolment procedures involved parental consent followed by participant assent and assessment for eligibility, we elected to additionally include some girls who reported sex in order to prevent stigmatization of girls, since their virginity could potentially be inferred by parents/others. We therefore randomly selected 26 schools from which we enrolled the first girl who reported ever having had sex, if her reported first sex was within the past year.

### Study procedures

The London School of Hygiene and Tropical Medicine Ethics Committee and the Medical Research Coordinating Committee, Tanzania, approved the study protocol in 2011. Consent procedures have been previously described. ^11^ Girls were enrolled between January and August 2012, and followed 3-monthly for 18 months. At each visit, girls had a face-to-face interview in Swahili with a female study nurse using a structured paper questionnaire, ^11^ and one nurse-assisted, self-administered vaginal Dacron swab was obtained, irrespective of reported sex. Girls who reported previous sex were offered a pregnancy test and asked about symptoms of reproductive tract infections at every visit. Those reporting symptoms were examined in the research clinic and offered syndromic treatment according to Tanzanian guidelines. At study completion, girls were offered a rapid test for HIV with appropriate referral if positive. In this paper, we present data from girls who reported passing sexual debut before or during the study.

### HPV detection and genotyping

Swabs were placed dry into cryotubes immediately after collection, stored in cold boxes with ice-packs in the field, submitted daily to the laboratory in Mwanza and stored at -20ºC. They were shipped to the Catalan Institute of Oncology, Barcelona, Spain, where HPV detection and genotyping were performed using the Linear Array HPV genotyping assay (Roche, USA). We used the updated HPV types from the International Human Papillomavirus (HPV) Reference Center ( www.hpvcenter.se ), and therefore reclassified HPV55 as HPV44, HPV64 as HPV34, and CP6108 as HPV89, and merged HPV-IS39 with HPV82. Therefore, 36 HPV genotypes were detected (HPV6, -11, -16, -18, -26, -31, -33, -34, -35, -39, -40, -42, -44, -45, -51, -52, -53, -54, -56, -58, -59, -61, -62, -66, -67, -68, -69, -70, -71, -72, -73, -81, -82, -83, -84, and -89). For this study, we classified HPV genotypes in IARC groups I (termed carcinogenic) and IIA (termed probably carcinogenic) as HR; HPV -16, -18, -31, -33, -35, -39, -45, -51, -52, -56, -58, -59, -68. All remaining genotypes were classified as LR. ^1^ Methods for DNA extraction, amplification and genotype detection were described previously. ^11^ Specimens negative for β-globin amplification were excluded, since vaginal sampling was assumed to be unsuccessful.

### Data management and statistical methods

Questionnaire data were double-entered into OpenClinica LLC (Akaza Research, MA, USA), and analysed using STATA V13.0 (StataCorp LP, TX, USA). Analyses were restricted to girls whose reported sexual debut was before enrolment or during follow-up ('sexually active'). Girls who were HIV-positive were excluded from all analyses. Further detail on statistical methods can be found in the Supplementary material (available as Supplementary data at *IJE* online).

For each HPV genotype, the number of prevalent infections (present at enrolment among those sexually active at entry), new infections (genotype not detected at enrolment or before reported sexual debut) and cleared infections (a new genotype that is no longer detected) was tabulated among all sexually active girls. The genotype-specific prevalence was estimated as the number of visits where the genotype was detected, divided by the number of sexually active visits.

Genotype-specific incidence was calculated; person-years (pys) at risk were calculated from enrolment (among girls whose reported sexual debut date was pre-enrolment) or date of sexual debut (among girls who reported sexual debut during follow-up). Kaplan-Meier methods were used to estimate time from sexual debut to first HPV infection among girls who reported sexual debut during follow-up and who were HPV-DNA negative at all visits before reported sexual debut ('HPV naïve').

The incidences of all new HPV, new HR-HPV and new LR-HPV infections were calculated among: (i) all sexually active girls; (ii) girls who reported sexual debut during follow-up; and (iii) HPV-naïve girls who reported sexual debut during follow-up. The overall incidence rate and 95% confidence interval (CI) were estimated using random effects Poisson regression to account for clustering of multiple infections within the same girl. Rate ratios (RR) for factors associated with the incidence of new HPV infections among all sexually active girls were estimated using random effects Poisson regression.

The genotype-specific clearance rate was calculated among all sexually active girls who had acquired a new genotype; pys at risk were calculated from the date of infection (midway between the last negative and first positive sample for the genotype). Kaplan-Meier methods were used to estimate the median and mean duration of genotype-specific infections and the proportion of infections cleared at 12 months. Cox regression with robust standard errors was used to examine risk factors for clearance.

## Results

### Cohort screening, enrolment and follow-up

We located 1177 (75.7%) of 1555 potentially eligible girls on the original school attendance lists. Of these, 801 (68.1%) met the age criteria, of whom 628 (78.4%) consented to be screened ( Supplementary Figure 1 , available as Supplementary data at *IJE* online). Of those screened, 503 (80.1%) were eligible and enrolled. Overall, 106 (21.1%) participants reported first sex: 29 at enrolment, 77 during follow-up. Among 29 girls whose reported date of first sex was before enrolment, median time from sexual debut to enrolment was 4.2 months (range 0.1-12.4). Among 106 girls reporting sex, 91 (85.8%) attended the final visit (18 months). The median [interquartile range (IQR)] follow-up time was 17.8 (17.4-17.9) months.

At the final visit, 49 of 91 (53.8%) participants accepted HIV testing and 1 (1.1%) was positive. The remaining 105 girls contributed 437 ‘sexually-active visits’ (visits after the reported date of sexual debut, including the enrolment visit) to the analysis; vaginal swabs were provided at 353 of these visits (80.8%), of which 323 (91.5%) were adequate specimens and were genotyped.

At enrolment, 71/105 (67.6%) participants were aged 16 years and the others were aged 15. Nearly two-thirds lived in rural areas (68, 64.8%);7 (6.7%) were in school; and over half were neither working nor schooling (60, 57.1%). During the study, 71 (67.6%) reported ever having cleansed inside their vagina, and only 1 girl reported being circumcised.

### HPV prevalence and incidence

**Figure 1.**
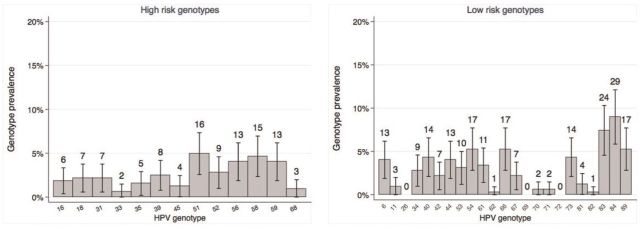
HPV genotype point prevalence (95% confidence interval), and number of infections at all visits after reported first sex in 105 adolescent girls. The HPV genotype-specific point prevalence was estimated as the number of visits where the genotype was detected, divided by the total number of visits after the reported date of sexual debut, including the enrolment visit. Visits with missing vaginal samples, or with samples that were β-globin negative, are excluded.

Of the 29 girls who reported ever having had sex at enrolment, 7 (24.1%) had at least one prevalent HPV infection at enrolment. A total of 28 new HR infections and 57 new LR infections were detected during follow-up ( Table 1 ). The most common HR genotypes were HPV51 (5.0% of visits), HPV58 (4.6%), HPV56 (4.0%) and HPV59 (4.0%) ( Figure 1 ).Genotype-specific incidence ranged from 2.2/100 person-years (pys) to 14.2/100 pys for each of the HR genotypes, and 0 to 24.1/100 pys for each of the LR genotypes ( Table 1 ). The highest incidence rates (per 100 pys) of HR types were for HPV58 (14.2), HPV51 (9.6) and HPV18 (6.7).

**Table 1.** HPV genotype prevalence, incidence, duration and clearance among 105 sexually active girls during follow-up

**HPV type**	**Prevalent (%)** ^a^	**New infections/pys (rate/100 pys)** ^b^	**New infections that were cleared (%)** ^c^	***N* cleared/pys (rate/100 pys) **	**Mean (median) months duration (Kaplan-Meier)** ^d^
**HigHRisk genotypes**					
HPV16	0	1/44.0 (2.3)	0	0/0.1 (0)	^‡^
HPV18	0	3/44.6 (6.7)	1 (33%)	1/0.5 (211.7)	2.7 (2.7)
HPV31	0	2/43.6 (4.6)	1 (50%)	1/0.9 (113.8)	6.1 (6.1)
HPV33	0	2/44.5 (4.5)	1 (50%)	1/0.4 (268.6)	3.0 (3.0)
HPV35	0	2/44.5 (4.5)	1 (50%)	1/1.0 (99.5)	6.0 * (4.9)
HPV39	1 (3%)	2/44.3 (4.5)	0	0/1.2 (0)	13.5 * ( ^†^ )
HPV45	0	1/43.1 (2.3)	1 (100%)	1/0.2 (403.6)	^‡^
HPV51	2 (7%)	4/41.7 (9.6)	3 (75%)	3/1.9 (158.7)	6.1 (6.1)
HPV52	0	1/44.3 (2.3)	1 (100%)	1/0.5 (198.5)	^‡^
HPV56	0	1/44.1 (2.3)	0	0/0.4 (0)	^‡^
HPV58	1 (3%)	6/42.1 (14.2)	0	0/1.8 (0)	13.2 * ( ^†^ )
HPV59	3 (10%)	2/42.7 (4.7)	1 (50%)	1/0.3 (289.9)	2.7 (2.7)
HPV68	0	1/44.9 (2.2)	0	0/0.1 (0)	^‡^
All HR infections ^e^	**7**	28	10 (36%)	10/9.4 (106.7)	6.9 * (6.0)
**Low-risk genotypes**					
HPV6	1 (3%)	5/41.0 (12.2)	2 (40%)	2/1.4 (146.8)	5.0 (6.1)
HPV11	0	1/45.1 (2.2)	0	0/0.2 (0)	^‡^
HPV26	0	0/45.5 (0)	–	–	–
HPV34	0	1/45.4 (2.2)	0 (0%)	0/0.1 (0)	^‡^
HPV40	0	2/43.8 (4.6)	0	0/0.3 (0)	1.7 * ( ^†^ )
HPV42	0	3/41.9 (7.2)	1 (33%)	1/0.8 (133.3)	6.1 (6.1)
HPV44	0	4/43.8 (9.1)	1 (25%)	1/1.3 (78.4)	6.2 * (4.9)
HPV53	0	2/42.7 (4.7)	1 (50%)	1/0.6 (180.4)	3.3 * (3.0)
HPV54	1 (3%)	4/41.8 (9.6)	1 (25%)	1/1.4 (73.6)	6.1 * ( ^†^ )
HPV61	1 (3%)	0/45.0 (0.0)	–	–	–
HPV62	0	3/43.6 (6.9)	1 (33%)	1/1.7 (59.2)	9.4 * (2.7)
HPV66	1 (3%)	6/41.5 (14.4)	2 (33%)	2/3.2 (62.5)	11.3 * (11.8)
HPV67	1 (3%)	1/44.6 (2.2)	1 (100%)	1/0.3 (299.4)	^‡^
HPV69	0	0/44.8 (0)	–	–	–
HPV70	0	0/44.5 (0)	–	–	–
HPV71	0	1/45.1 (2.2)	0	0/0.4 (0)	^‡^
HPV72	0	0/45.5 (0)	–	–	–
HPV73	1 (3%)	4/43.1 (9.3)	1 (25%)	1/0.9 (117.6)	3.5 * (2.7)
HPV81	0	1/45.0 (2.2)	0	0/0.1 (0)	^‡^
HPV82	0	0/45.4 (0)	–	–	–
HPV83	0	3/40.7 (7.4)	1 (33%)	1/2.0 (50.6)	11.9 * ( ^†^ )
HPV84	2 (7%)	9/37.3 (24.1)	3 (33%)	3/3.1 (97.5)	6.0 * (6.0)
HPV89	0	7/43.6 (16.1)	1 (14%)	1/1.8 (55.6)	7.0 * (4.9)
All LR infections ^e^	8	57	16 (28%)	16/19.4 (82.6)	8.9 * (6.1)
**All HPV infections**	**15** ^±^	**85**	**26 (31%)**	**26/28.8 (90.4)**	**8.4** * **(6.1)**

^a^ Positive for that genotype at the enrolment visit, among 29 girls who were sexually active at enrolment.

^b^ New infection defined as first positive test for the specific HPV type, among those not infected at enrolment or before reported sexual debut. Girls with gaps > 180 days in observation time are censored at the most recent available HPV result before the gap.

^c^ Clearance defined as ≥ 2 consecutive samples negative for the specific genotype; denominator is total genotype-specific new infections.

^d^ Mean duration of new infections estimated using Kaplan-Meier methods restricted by the longest follow-up time (i.e. duration).

^e^ Total number of group (HR or LR)-specific infections among 105 girls.

*Mean duration of infection for the genotype is underestimated because the individual with the longest observed duration was censored.

^†^ Median duration could not be estimated because survival curve does not drop below 50%.

^‡^ One infection only, Kaplan-Meier survival function not estimated.

^±^ 15 infections within 7 girls: 5 had at least one HR HPV genotype at enrolment, 5 had at least one LR HPV genotype at enrolment, 7 had any genotype at enrolment.

**Figure 2.**
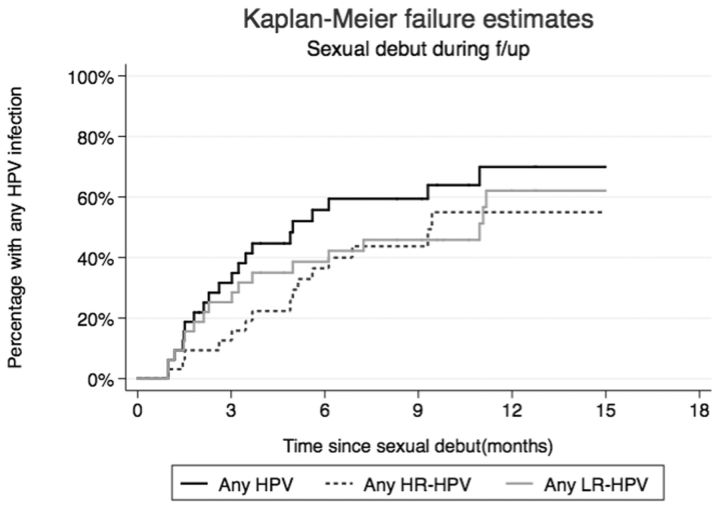
Time from sexual debut to first infection with any HPV, any HR HPV or any LR HPV, among 41 girls who reported sexual debut during follow-up and were HPV-naïve at time of reported sexual debut. Kaplan Meier curves are calculated separately for each HPV group.

Among the 76 girls who reported first sex during follow-up, 35 (46.1%) had at least one HPV infection detected before the reported date of first sex. Among HPV-naïve girls, median time from reported sexual debut to HPV infection was 4.9 months ( Figure 2 ), and to first HR-HPV was 9.3 months. Cumulative incidence of any HPV infection at 6 months was 52.8%: 35.8% for HR and 34.7% for LR genotypes.

The overall incidence rate (per 100 pys) of new HPV infections in sexually active girls ( Table 2 ) was 225 (95% CI: 166-305); HR-HPV incidence was 66 (95% CI: 45-95) and LR-HPV was 157 (95% CI: 111-222). Among girls who reported sexual debut during follow-up, the incidence rate for new HPV infections and for new HR-HPV infections were 209 (95% CI: 146-299) and 63 (95% CI: 40-99), respectively. Restricting to HPV-naïve girls, these were 193 (95% CI: 118-316) and 72 (95% CI: 42-122), respectively. Overall, HPV was detected in 46% of ‘sexually active visits’ in all girls ( Table 2 ).

**Table 2.** Incidence and point prevalence of HPV in adolescent girls who reported sex

**Outcome**	** All girls who reported sexual debut before or during the study ( *N* = 105) **	** All girls who reported sexual debut during study ( *N* = 76) ** ^a^	** Girls who reported sexual debut during study and were HPV-naïve ( *N* = 41) ** ^b^	** Reported sexual debut prior to enrolment ( *N* = 29) **
**Incidence**				
	**New infections/person-years (rate/100 person-years, 95% CI)** ^c^			
All HPV	119/56.4 (225; 166-305)	62/30.1 (209; 146-299)	40/19.5 (193; 118-316)	57/26.3 (248; 144-425)
All HR HPV	37/56.4 (66; 45-95)	19/30.1 (63; 40-99)	14/19.5 (72; 42-122)	18/26.3 (71; 37-135)
All LR HPV	82/56.4 (157; 111-222)	43/30.1 (146; 97-218)	26/19.5 (127; 66-246)	39/26.3 (176; 95-327)
**Prevalence**				
	**Total infections (number of visits with at least one infection/sexually active visits; % of all visits)** ^d^			
All HPV	323 (148/323; 45.8%)	186 (87/172; 50.6%)	91 (40/106; 37.7%)	137 (61/151; 40.4%)
All HR HPV	108 (87/323; 26.9%)	57 (49/172; 28.5%)	32 (26/106; 24.5%)	51 (38/151; 25.2%)
All LR HPV	215 (118/323; 36.5%)	129 (70/172; 40.7%)	59 (29/106; 27.4%)	86 (48/151; 31.8%)

^a^ HPV incidence among all girls who reported passing sexual debut during the study; includes 35 girls in whom HPV was detected before reported sexual debut (infections before reported sexual debut do not contribute to the incidence estimate in this column, but girls are not excluded from the analysis).

^b^ HPV incidence among 41 girls who reported passing sexual debut during the study and no HPV was detected before reported sexual debut.

^c^ Rate estimated from random effects Poisson regression: point estimates and 95% CI take into account correlation of repeated infections within girls. Girls assumed to be continually at risk and can acquire > 1 infection at each visit. Observation time after gaps > 180 days contributes to the analysis, therefore total number of infections is different from that in Table 1 .

^d^ Total number of genotype-specific infections and number of visits where at least one genotype was detected at all visits after reported date of sexual debut, including enrolment visit.

### Risk factors for incidence of new HPV infection

In the adjusted analysis ( Table 3 ) there was evidence of an association with: not being in a regular job or training as compared with those with an occupation [adjusted (a) RR = 1.95, 95% CI: 1.1-3.42], with the reporting of recent sex (aRR 2.48, 95% CI: 1.40-4.37) and having known the most recent partner for longer (aRR 3.15, 95% CI: 1.32-7.50). There was weak evidence of a higher rate of new HPV infections among girls reporting three or more partners compared with only one partner, and weak evidence of a lower rate among girls who reported vaginal cleansing (aRR 0.69, 95% CI: 0.43-1.10).

**Table 3.** Association of selected potential risk/protective factors ^a^ with any new HPV infection among 105 adolescent girls who reported previous sex

	**Number of infections** ^b^ **/person-years (rate/100 pys)**	**Crude RR (95% CI)**	**Adjusted RR (95% CI)** ^c^
**Sociodemographic (enrolment)**			
Age at enrolment		*P* = 0.45	
15 years	32/19.1 (189)	1	
16 years	87/37.3 (244)	1.29 (0.67-2.47)	
Religion		*P* = 0.83	*P* = 0.83
Christian	99/49.2 (213)	1	1
Muslim	16/5.5 (333)	1.56 (0.59-4.12)	1.57 (0.60-4.16)
Other	4/1.7 (219)	1.16 (0.11-12.30)	1.07 (0.10-11.30)
Socioeconomic status score (tertiles)		*P* = 0.57	*P* = 0.63
Low	58/25.5 (246)	1	1
Middle	33/20.3 (174)	0.70 (0.35-1.43)	0.72 (0.36-1.48)
High	28/10.6 (256)	1.04 (0.48-2.23)	1.03 (0.48-2.21)
**Sociodemographic (time-varying)**			
Current residence		*P* = 0.80	*P* = 0.53
Urban	50/20.7 (235)	1	1
Rural	65/34.6 (217)	0.92 (0.49-1.72)	0.82 (0.44-1.53)
Current occupation		*P* = 0.05	***P* = 0.06 **
School	5/2.9 (164)	1.13 (0.30-4.26)	**1.15 (0.30-4.36)**
Work | vocational training	29/23.1 (144)	1	**1**
Not working	85/30.4 (283)	1.96 (1.12-3.43)	**1.95 (1.11-3.42)**
Currently married		*P* = 0.66	*P* = 0.79
No	90/43.9 (217)	1	1
Yes	29/12.5 (248)	1.14 (0.63-2.09)	0.92 (0.50-1.70)
Alcohol since last visit		*P* = 0.42	*P* = 0.48
No	116/55.2 (228)	1	1
Yes	3/1.2 (134)	0.59 (0.16-2.22)	0.62 (0.16-2.40)
**Behavioural (time-varying)**			
Total partners ever		*P* = 0.09	*P* = 0.09
1	86/43.1 (225)	1	1
2	16/10.0 (155)	0.69 (0.35-1.34)	0.77 (0.38-1.54)
3+	16/2.5 (509)	2.26 (0.83-6.17)	2.76 (0.95-8.04)
Number of times had sex in past 3 months		*P* = 0.005	***P* = 0.008 **
0	49/31.4 (159)	1	**1**
1	30/9.3 (400)	2.52 (1.44-4.42)	**2.48 (1.40-4.37)**
2+	39/15.5 (257)	1.62 (0.95-2.77)	**1.52 (0.88-2.63)**
Most recent male sexual partner circumcised		*P* = 0.22	*P* = 0.23
No	33/16.1 (182)	1	1
Yes	80/31.5 (263)	1.47 (0.79-2.72)	1.48 (0.77-2.85)
Don't know ^d^	5/8.0 (109)	–	–
Most recent sexual partner was in a concurrent relationship		*P* = 0.06	*P* = 0.25
No	53/31.1 (171)	1	1
Yes	12/2.7 (402)	2.37 (0.99-5.68)	1.83 (0.66-5.07)
Don't know ^d^	53/21.7 (287)	–	–
Age difference of most recent partner		*P* = 0.11	*P* = 0.10
≤ 2 years	8/8.9 (82)	1	1
3–5 years	26/11.0 (215)	2.65 (1.03-6.83)	(1.04-6.45)
>5 years	35/13.1 (237)	2.64 (1.04-6.73)	2.33 (0.94-5.77)
Don't know ^d^	49/22.6 (316)	–	–
Used condom at most recent sex		*P* = 0.93	*P* = 0.90
No	105/46.5 (226)	1	1
Yes	13/9.1 (235)	1.04 (0.45-2.40)	0.95 (0.41-2.20)
Does partner put saliva on penis		*P* = 0.58	*P* = 0.26
No	78/33.5 (229)	1	1
Yes	7/1.7 (309)	1.34 (0.48-3.75)	1.87 (0.64-5.48)
Don't know ^d^	2/0.5 (292)	–	–
Does partner use vaseline for sex		*P* = 0.007	*P* = 0.17
No	77/35.7 (208)	1	1
Yes	10/1.1 (717)	3.44 (1.47-8.04)	2.06 (0.76-5.63)
Time had known most recent partner before first sex (with that partner)		*P* = 0.14	**P = 0.03**
< 1 month	26/16.1 (143)	1	**1**
1-6 months	64/28.0 (252)	1.76 (0.89-3.47)	**1.76 (0.90-3.46)**
6+ months	28/11.0 (314)	2.19 (0.92-5.20)	**3.15 (1.32-7.50)**
Cleansed vagina in past 3 months ^e^		*P* = 0.02	*P* = 0.11
No	68/29.8 (300)	1	1
Yes	51/26.6 (169)	0.56 (0.35-0.90)	0.69 (0.43-1.10)

^a^ Potential risk/protective factors were examined using a conceptual framework with three levels; age was considered an a priori confounder and included in all models. Age-adjusted sociodemographic factors at enrolment were retained in a core model if associated with HPV infection at *P* < 0.10. Time-varying sociodemographic factors were added sequentially and retained if associated at *P* < 0.10. Time-varying behavioural factors were then added sequentially, and retained at *P* < 0.10. All *P* -values presented in the table are from the likelihood ratio test.

^b^ Girls are assumed to be continually at risk and can acquire > 1 infection at each visit. Observation time after gaps > 180 days contributes to the analysis; therefore, the total number of infections (119) is different from that in Table 1 .

^c^ Sociodemographic factors at enrolment adjusted for age (a priori). Time-varying sociodemographic factors adjusted for age (a priori) and all independent sociodemographic predictors of HPV infection (at *P* < 0.1) (occupation). Behavioural factors adjusted for age, occupation and all independent behavioural predictors of HPV infection (number of times had sex in past 3 months and time knew most recent partner before sex (variables in bold).

^d^ ‘Don’t know’ responses considered missing data and not included in analysis.

^e^ Vaginal cleansing is cleaning inside the vagina with water, soap or other products using fingers or a cloth.

### HPV duration and clearance

During follow-up, 33 girls acquired at least one new HPV genotype and contributed 85 new infections to the genotype-specific duration and clearance analysis. In total, 26 of 85 (30.6%) new infections were cleared during follow-up. Median duration of new HPV genotype-specific infections was 6.1 months. This was 6.0 and 6.1 months for new HR and new LR-HPV genotypes, respectively. Overall rate of clearance (per 100 pys) was 90.4 for any HPV genotype. After adjustment for age, there were no significant associations with any examined factors ( Table 4 ).

**Table 4.** Clearance of new HPV infections ^a^ and associated factors among girls who reported having had sex at any time during follow-up (unit of analysis is the infection)

	**Number cleared/person-years (rate/100 pys)**	**Crude HR (95% CI)**	**Adjusted HR (95% CI)**
**Sociodemographic (enrolment)**			
Age at enrolment		*P* = 0.03	
15 years	8/6.1 (132.2)	1	
16 years	18/22.7 (79.3)	0.09 (0.01-0.74)	
Religion		*P* = 0.08	*P* = 0.21
Christian	25/23.1 (108.3)	1	1
Muslim	1/4.8 (20.8)	0.39 (0.13-1.13)	0.53 (0.20-1.43)
Other	0/0.9 (0.0)	–	–
Socioeconomic status score (tertiles)		*P* = 0.10	*P* = 0.12
Low	16/15.3 (104.6)	1	1
Middle	7/8.0 (87.6)	0.81 (0.22-3.02)	0.73 (0.37-1.45)
High	3/5.5 (54.9)	0.35 (0.13-0.93)	0.40 (0.14-1.09)
**Sociodemographic (time-varying)**			
Current residence		*P* = 0.10	*P* = 0.24
Urban	13/15.2 (85.4)	1	1
Rural	13/12.9 (101.0)	2.23 (0.85-5.85)	0.44 (0.11-1.71)
Current occupation		*P* = 0.88	*P* = 0.56
School	6/7.8 (77.0)	1	1
Work | vocational training	0/0.1 (0.0)	–	–
Not working	20/20.8 (96.0)	0.90 (0.24-3.38)	1.55 (0.34-7.09)
Currently married		*P* = 0.25	*P* = 0.46
No	21/23.8 (88.3)	1	1
Yes	5/5.0 (100.3)	0.44 (0.11-1.78)	0.60 (0.15-2.33)
Alcohol since most recent visit		P = 0.35	*P* = 0.35
No	24/27.5 (87.2)	1	1
Yes	2/1.2 (164.0)	2.45 (0.37–16.28)	2.45 (0.37-16.28)
**Behavioural (time-varying)**			
Total partners ever		*P* = 0.58	*P* = 0.16
1	18/17.6 (102.6)	1	1
2	4/5.4 (73.8)	0.84 (0.17-4.05)	0.26 (0.04-1.53)
3+	4/5.7 (70.7)	0.60 (0.23-1.57)	0.84 (0.35-2.01)
Number of times had sex in the past 3 months		*P* = 0.14	P = 0.14
0	13/11.0 (118.4)	1	1
1	0/6.2 (0.0)	–	–
2+	13/11.5 (112.7)	0.21 (0.02-1.69)	0.21 (0.02-1.69)
Most recent male sexual partner was circumcised		*P* = 0.06	*P* = 0.18
No	15/13.7 (109.7)	1	1
Yes	11/14.4 (76.3)	0.38 (0.14-1.04)	0.56 (0.24-1.30)
Don't know ^b^	0/0.5 (0.0)	–	–
Most recent sexual partner was in concurrent relationship			
No	17/18.2 (93.3)	–	–
Yes	0/1.6 (0.0)	–	–
Don't know ^b^	9/8.8 (102.1)	–	–
Used condom at last sex			
No	26/26.9 (96.8)	–	–
Yes	0/1.8 (0.0)	–	–
Partner put saliva on penis		*P* = 0.75	*P* = 0.75
No	18/22.0 (81.8)	1	1
Yes	3/2.0 (150.7)	0.88 (0.40-1.92)	0.88 (0.40-1.92)
Don't know ^b^	–	–	–
Partner used vaseline for sex			
No	21/23.0 (91.3)	–	–
Yes	0/1.0 (0.0)	–	–
Time had known most recent partner before sex		*P* = 0.13	*P* = 0.89
< 1 month	7/7.4 (94.9)	1	1
1-6 months	17/17.2 (98.6)	2.05 (0.64-6.53)	0.98 (0.30-3.15)
6+ months	2/3.8 (52.1)	0.82 (0.17-4.01)	0.72 (0.09-5.54)
Cleansed vagina in past 3 months ^c^		*P* = 0.02	*P* = 0.30
No	19/15.9 (119.4)	1	1
Yes	7/12.8 (54.5)	0.26 (0.08-0.83)	0.49 (0.13-1.87)

^a^ New infection defined as first positive test for the specific HPV type, among those not infected at enrolment or before reported sexual debut. Girls with gaps >180 days in observation time are censored at latest available HPV result before the gap. All *P* -values are from likelihood ratio tests.

^b^ ‘Don’t know’ responses considered missing data and not included in analysis.

^c^ Vaginal cleansing is cleaning inside the vagina with water, soap or other products using fingers or a cloth.

## Discussion

In this study, we demonstrate an extremely high incidence of vaginal HPV infection after first sex in adolescent Tanzanian girls. Acquisition was rapid in the initial months after first reported sex, and over half of the girls were positive for any HPV DNA in these first 6 months. These findings support current recommendations that adolescent girls should ideally be vaccinated before first sex. ^13^

Few studies have examined HPV incidence in young women after sexual debut. First acquisition of HPV (which predominantly occurs in the months after first penetrative sex) is a unique opportunity to document HPV genotypes to which young women are exposed and which may then become latent (and therefore un-detectable) until reactivation later in life. Current molecular testing cannot differentiate reactivation from first acquisition or re-infection and therefore all studies of HPV incidence in sexually active women can only record presumed incidence of HPV infections, since some apparent new infections may actually be re-activations. HPV84, -83, -61, -66 and CP-108 were the most common genotypes seen in our study. This is in contrast to global prevalence data in cytologically normal women that have reported HPV16, -18, -52, -31 and -58 as the most prevalent genotypes. ^6^ In our study the incidence rate of HPV vaccine genotypes was low, ranging between 2.4 and 13.6 per 100 pys for each of the HPV types covered by the quadrivalent vaccine (HPV6, -11, -16 and -18); and between 1.3 and 13.6 per 100 pys for each of the HPV types covered by the new nonavalent vaccine (HPV6, -11, -16, -18, -31, -33, -45, -52, -58).Incidence rates of HPV16 (2.3/100 pys) and HPV-18 (6.7/100 pys) were lower relative to other genotypes. Our data could be used in modelling studies to explore whether catch-up vaccination campaigns in older girls (for example up to age 17 years) have additional impact on cervical cancer incidence.

The overall HPV incidence in our study (187/1000 person-months) was far higher than that reported in already sexually active women. A cohort study of sexually active women in Brazil, median age 33 years, reported an incidence of 13.4/1000 person-months, ^14^ and a study in women in Canada, median age 21, reported an incidence of 19/1000 person-months. ^15^ Cumulative incidence has been reported as 39-44% at 24 to 36 months after first sex in Brazil and the USA, ^7^^,^^8^^,^^14^ lower than 53% at the much shorter follow-up period of 6 months in our study. Young women are known to have a high incidence of infection, but the particularly high incidence in our study may be driven by a high HPV prevalence in the male partners of these young women. ^6^^,^^7^^,^^16–18^ However, the incidence in our cohort is higher than in other studies in young women in East Africa: in sexually active women in Uganda (median age 20 years), HPV incidence was 30.5/100 pys, ^19^ and 74/100 pys in women in Mwanza, Tanzania. ^17^ The latter study was performed in the same region, but participants were older (median age 18), and all had reported previous sex. These comparative findings support the suggestion that incidence is highest around the time of first sex.

Comparing the incidence of individual genotypes in our Tanzanian study with a study in women in the USA aged 16-23 years, 5% of whom reported never having had sex ^20^ : in our participants, HPV6, -11 and -18 incidences were 3-fold higher. However, a lower rate was seen for HPV16 in our study (2.3/100 pys) compared with the study in the USA (5.4/100 pys). This is in keeping with findings that HPV-16 is less common in SSA than in other regions including the USA. ^6^^,^^21^

Not working was associated with increased HPV incidence compared with being employed or in vocational training. Girls not working may be at increased risk of engaging in sex in exchange for gifts or money or of forced sex, which are risk factors for HIV and other STIs, ^22^ but have not clearly been identified as risk factors for HPV. ^23^^,^^24^ These behaviours were infrequently reported in our study, although they have been described in local studies in older women. ^25^ Knowing a partner for 6 or more months before sex was associated with a more than 3-fold risk of incident HPV compared with knowing a partner for under 1 month. Girls may be more likely to be involved in risky sex (i.e. without a condom) and therefore be at increased risk of HPV, ^26^ if a partner is well-known to them. Contrary to that, reported condom use at most recent sex was not associated with lower HPV incidence, although numbers were small. Reported male partner circumcision was similarly not associated with incident HPV, in contrast to a large study in Uganda. ^27^ However, girls in our study may not have known whether their partners were or were not circumcised.

Limitations of our study include the use of self-administered swabs rather than clinician-collected cervical swabs. We used self-administered swabs since speculum examination was undesirable in girls who had not passed sexual debut. Over 90% were β-globin positive, indicating adequate sampling. ^28^ Further, a previous study in Uganda demonstrated good HPV-genotype correlation in self-administered and clinician-administered swabs. ^29^

Unobserved intervals (without vaginal swab results) of over 180 days were removed from the analysis. However, sensitivity analyses, where girls were assumed to be uninfected with a new genotype during those intervals, gave similar results [incidence among all ‘sexually active’ girls was 158/100 pys, (95% CI: 123–203)]. Samples negative or missing for a given genotype, but which had been taken between two samples positive for that genotype, were classified as positive since studies describing long-term persistence have demonstrated sporadic detection of the same genotype early in the course of a persistent infection. ^30^ We excluded one girl who was HIV-positive at study completion, since HPV incidence is higher with HIV infection. ^31^^,^^32^ Only 46% of participants attending the final visit accepted an HIV test; therefore HIV-positive girls may have been included in the analysis. However, national estimates indicate a very low HIV prevalence in 15-19-year-old girls in Tanzania (1.3%). ^33^

Median time from first reported sex to acquisition of any HPV was 5 months. This is longer than 2.4 months reported in college students in the USA tested 3-monthly. ^34^ Differences in the types of relationships formed (marriage vs casual sex partner), recent sex and condom use may explain these differences, since some of these have been identified as risk factors for acquisition in our or other studies. ^26^^,^^35^ Reporting bias may have influenced accurate assessment of these risks: participants in our study may have been less willing to report sex and had less accurate recall of dates of sex compared with women in the USA study. The median duration of infection in our study was shorter (6 months) than in previous studies (reported range 8-31 months ^14^^,^^15^^,^^36^ ). This may be an underestimate since the duration of follow-up was limited compared with these previous studies, and was dependent on the point at which girls reported sexual debut. ^14^^,^^15^^,^^36^ Clearance events may have been falsely observed through lack of detection of HPV due to self-sampling. As discussed earlier, the presence of β-globin was considered necessary to ensure adequate vaginal sampling and will have reduced this risk. A short duration of infection could be due to cervico-vaginal immune activation in Tanzanian girls, which has been shown to be higher in STI- and HIV-uninfected young women in Kenya compared with the USA. ^37^ High levels of endocervical T lymphocytes identified in those women in Kenya could have mediated HPV clearance. ^37^ Finally, higher cervical HPV viral load, age over 30 years, being HIV-positive and having a high number of sex partners were associated with lower HPV clearance in women in Uganda. ^32^ We identified no associations with HPV clearance, potentially because our cohort displayed little variation in age or number of sex partners, and girls were either HIV-negative or of unknown HIV status.

We report a rapid acquisition of HPV infection, extremely high incidence and rapid clearance in young women after their first reported sex. This study was carried out in a region with one of the highest incidences of cervical cancer in the world, and our findings may help to explain these high rates of cervical cancer and the high HPV prevalence observed in East Africa ^6^ and support the current recommendation that HPV vaccination should be given to girls before their first sex. ^38^

## Funding

This work was supported by the Wellcome Trust [grant number ITCRBE30] and the WHO Collaborating Centre for HIV Surveillance in Zagreb, Croatia, via a grant from the Croatian Ministry of Foreign and European Affairs. K.B. and R.J.H. receive support from Medical Research Council (MRC) and Department for International Development (DFID) [grant number G0700837].

## Supplementary Material

Supplementary Data
