# Supplementary material for: Rapid acquisition of HPV around the time of sexual debut in adolescent girls in Tanzania
Source: Int J Epidemiol. 2016 Mar 4;45(3):762–73. doi: 10.1093/ije/dyv367 (PMC5005945; doi:10.1093/ije/dyv367)
Supplement: Supplementary Data [file dyv367_supplementary_data.zip › ije-2015-07-0864-File005.pptx]

## Slide 1
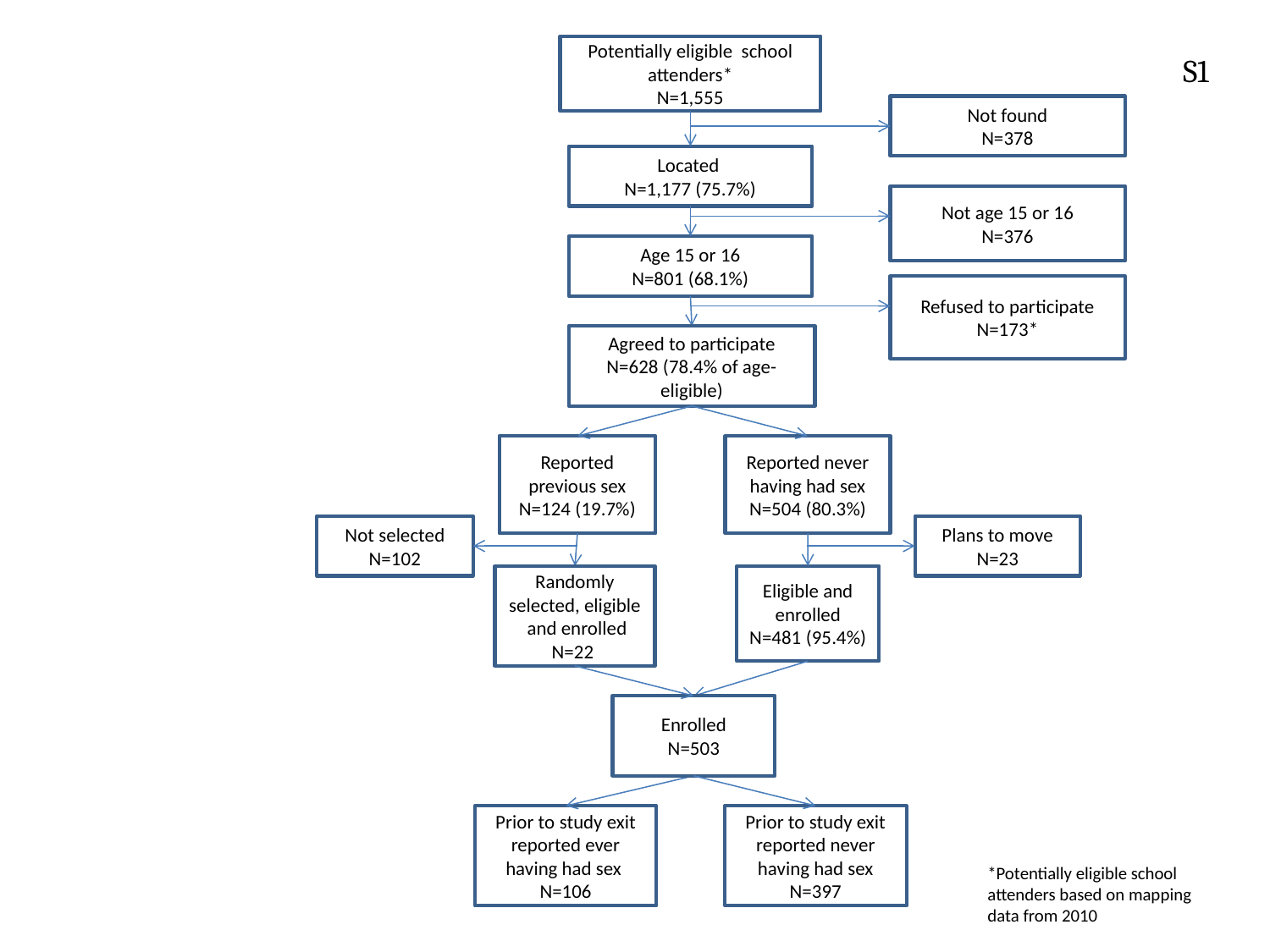

Potentially eligible school attenders*
N=1,555
Not found
N=378
Located
N=1,177 (75.7%)
Not age 15 or 16
N=376
Age 15 or 16
N=801 (68.1%)
Refused to participate
N=173*
Agreed to participate
N=628 (78.4% of age-eligible)
Reported previous sex N=124 (19.7%)
Reported never having had sex N=504 (80.3%)
Not selected
N=102
Plans to move
N=23
Eligible and enrolled
N=481 (95.4%)
S1
Enrolled
N=503
Randomly selected, eligible and enrolled
N=22
Prior to study exit reported ever having had sex
N=106
Prior to study exit reported never having had sex
N=397
*Potentially eligible school attenders based on mapping data from 2010
