# Supplementary material for: Rapid acquisition of HPV around the time of sexual debut in adolescent girls in Tanzania
Source: Int J Epidemiol. 2016 Mar 4;45(3):762–73. doi: 10.1093/ije/dyv367 (PMC5005945; doi:10.1093/ije/dyv367)
Supplement: Supplementary Data [file dyv367_supplementary_data.zip › ije-2015-07-0864-File004.docx]

**Supplementary information**

**Statistical methods**

A detailed analysis plan was produced before data analysis. For genotype-specific incidence, the date of infection was defined as the midpoint between the last negative and first positive sample for that genotype. Since the incidence rate was very high and most infections were of short duration, girls with periods longer than 180 days with missing HPV results were censored at the date of the last available HPV result before the interval with missing data.

For the analyses of overall incidence of all new HPV infections, all new HR HPV, and all new LR HPV, girls were assumed to be continually at risk of infection with a new genotype, and could acquire more than one new infection at each visit. Periods longer than 180 days with missing HPV results were treated as gaps in the observation time and removed from the analysis; however, observation time after the gap contributed to the analysis.

For the analysis of factors associated with the incidence of new HPV infections among all sexually active girls, we used a conceptual framework with three levels. Age was considered an a priori confounder and was included in all models. Socioeconomic status was measured using an asset index, created by combining data on ownership of common household items in the entire cohort (i.e. including non-sexually active participants) using principal component analysis. Age-adjusted sociodemographic factors at enrolment were retained in a core model if associated with HPV infection at p<0.10. Time-varying sociodemographic factors were added to this core model sequentially and retained if associated at p<0.10. Time-varying behavioural factors were then added sequentially, and retained if they remained associated at p<0.10. This strategy allowed us to assess the effects of variables at each level of the framework, adjusted for more distal variables.

Clearance of a genotype-specific HPV infection was defined as two consecutive negative samples, or one negative and one missing sample, for the genotype. In situations where a girl tested negative for a genotype between two positive samples for the same genotype, the intervening negative was considered to be a false negative. The date of clearance was defined as the midpoint between the last positive and first negative sample. Girls who did not clear an infection were censored at the date of their last sample.

Factors associated with clearance of a new HPV infection were examined using methods for multiple failure-time data. The unit of analysis was the HPV infection; therefore, girls infected with multiple genotypes could clear more than one infection. Failure (i.e. clearance) events were assumed to be unordered, so clearance of a genotype-specific infection was independent of clearing other genotypes. Cox regression was used to examine risk factors for clearance. The Cox model was stratified by HPV genotype, so that each HPV genotype was allowed to have a different baseline hazard function. Robust standard errors were used to adjust for correlation of repeated clearance events among girls.
